# Effects of Corn–Soybean Strip Intercropping on Control Efficiency of Insect Pests and Crop Yields

**DOI:** 10.3390/plants14213358

**Published:** 2025-11-02

**Authors:** Xiping Wei, Zhoulong Cheng, Junjie Wang, Chongyi Liu, Shanglin Yang, Fajun Chen

**Affiliations:** 1 State Key Laboratory of Agricultural and Forestry Biosecurity, Department of Entomology, College of Plant Protection, Nanjing Agricultural University, Nanjing 210095, China; 2024202066@stu.njau.edu.cn (X.W.); chengzhoulong@stu.njau.edu.cn (Z.C.); liuchongyi@stu.njau.edu.cn (C.L.); 2022802167@stu.njau.edu.cn (S.Y.); 2 College of Agriculture, Nanjing Agricultural University, Nanjing 211800, China; 11121203@stu.njau.edu.cn

**Keywords:** corn–soybean strip intercropping, insect pests, population dynamics, community diversity, yields, *LER*, *CR*

## Abstract

Corn–soybean strip intercropping (abbr. CSSI system) can enhance species biodiversity and ecological services for ecological control of insect pests. To improve its effectiveness and fully utilize it to improve ecological control of insect pests and crop production, two monoculture types of corn (C) and soybean (S), and two strip intercropping patterns (i.e., C3S3 and C3S4, indicating three rows of corn strip intercropped with three and four rows of soybeans respectively), were conducted to assess the CSSI system’s (i.e., C3S3 and C3S4) impacts on the abundance of insect pests and crop yields by a two-year field experiment. The results indicated that a total of 11 species of insect pests were found in the CSSI system. Compared with C or S monoculture, the community indexes of insect pests (including the Shannon–Wiener diversity index (*H*), the Pielou’s evenness index (*E*), and the Margalef’s richness index (*D*)) increased, and the Simpson’s dominance index (*C*) decreased in the C3S3 and C3S4 patterns in 2022. Compared to the C and S monoculture, the CSSI system decreased the population dynamics of total insect pests and the key insect pests *Trialeurodes vaporariorum* on corn and soybean plants, respectively. In the CSSI system, *T. vaporariorum* exhibited higher population dynamics on corn plants than on soybean plants, indicating a preference for corn plants under the CSSI system. Moreover, the corn yield per hectare in the C3S4 pattern was significantly higher than that of the C monoculture in 2022–2023. The biomass per plant and the 1000-grain weight of corn in the C3S3 pattern were significantly lower than that in the C monoculture and C3S4 pattern in 2022. The biomass per plant, the 1000-grain weight and yield per hectare of soybean in the C3S3 and C3S4 patterns were significantly lower than that in the S monoculture in 2022–2023. The land equivalent ratio (*LER*) was <1.0 in the CSSI system, posing yield loss risk for soybeans in the CSSI system. The competitive ratio (*CR*) of corn was greater than soybean in the CSSI system. In addition, the yield of corn and soybeans were not significantly correlated with the abundance of total insect pests, while the soybean yield was significantly positively correlated to the abundance of *T. vaporariorum*. In conclusion, it is presumed that the CSSI system can decrease the abundances of insect pests, particularly key insect pests, and maintain their community stability, thereby preventing insect pests’ outbreak. However, the CSSI system is disadvantageous for soybean yield, as it cannot fully utilize land resources and may pose a risk of system yield loss.

## 1. Introduction

Crop diversity in farmlands has rapidly declined as a result of agricultural monoculture intensification [1,2,3]. Over the past six decades, monoculture intensification has been widely adopted across the globe, especially focusing on four major crops: wheat (*Triticum aestivum* L.), rice (*Oryza sativa*), corn (*Zea mays* L.) and soybean (*Glycine max* L.). It has seriously weakened crop biodiversity, and reduced both the abundance of natural enemies and suppressive effects on insect pests, which increase the risk of insect pest outbreaks [3,4,5,6]. Intercropping involves two or more crop species planted in the same farmland system during the whole or part of a growing season, which is an effective strategy to increase crop biodiversity and enhance natural control of pests [2,3]. Rakotomalala et al. (2023) did a meta-analysis of 63 articles from 18 countries, and found that intercropping significantly increased the overall abundance of beneficial arthropods by 36% and species richness by 27%, while reducing by 38% of the abundance and 41% of the density of arthropod pests [3]. Regarding spatial arrangement, strip intercropping is an effective strategy to increase crop biodiversity and enhance biocontrol compared to mixed and relay intercropping [1,2,3]. Intercropping can enhance conservation biological control providing both food and shelter that attract and sustain natural enemies, as well as producing different chemical volatiles that influence the behavior of predators and pests [7,8,9,10,11]. And selecting crop combinations is particularly critical in strip intercropping for biocontrol. Cereal–legume intercropping outperformed cereal–cereal monoculture in ecological control of insect pests [3,12,13].

Corn and soybean are two of the most important crops worldwide. Corn is widely cultivated due to its adaptability to diverse climates and soils, making it a staple in many regions [14]. Soybean stands out for its nitrogen-fixing ability, which reduces its reliance on soil nitrogen and enables it to be cultivated in various soil types [15]. As a result of agricultural monoculture, the outbreak of pests has a great impact on corn and soybean production in fields. Recent research indicated that about 40 species of pests decreased the quality and yield of corn [16]. Soybean yield loss ranged between 25.8% and 42.8% due to insect pests [17]. So, prevention and control of insect pests of corn and soybean crops are very important. Corn–soybean strip intercropping (abbr. CSSI) is a sustainable farming practice, which can help to restore or maintain biodiversity and associated ecosystem services, widely used in China [5,18,19]. The CSSI system could increase insect biodiversity, which in turn affects the “push-pull” process resulting from top-down and bottom-up mechanisms of natural pest control, thereby playing a role in the management of target pests [11,18,20,21,22]. Fattah et al. (2023) showed that corn–soybean intercropping could be the best practice for sustainable agriculture production due to facilitating the increase of growth of predator populations and reducing the level of crop damage due to pest attacks [22]. Currently, the CSSI system primarily utilizes row ratios of 1:1, 2:2, 2:3, 2:4, and 2:6 [5,19,23,24,25]. As corn and soybean were sown in different rows, pest control effectiveness was variable [19,26]. Specifically, among the intercropping patterns with rows of corn to soybean as 2:2, 2:3, and 2:4, the 2:4 intercropping pattern was the most beneficial for pest control, which reduced the abundance of insect pests on soybean plants [19]. Li et al. (2019) indicated that the Shannon–Wiener diversity index (*H*), the Pielou’s evenness index (*E*), and the Margalef’s richness index (*D*) of insects in the CSSI system (row ratios of corn and soybean as 1:2, 1:4, and 1:6) were higher than those in the monoculture, and the Simpson’s dominance index (*C*) was lower than that in the monoculture [27]. Compared with the soybean monoculture, Hafid et al. (2021) proved that the C3S3 and C3S4 patterns significantly reduced the average population of *Nezara viridula* and *Bemisia tabaci* [28]. Lisdayanti et al. (2022) showed that the CSSI system significantly affected the abundance and number of natural enemies species [29]. The C5S1 pattern had the highest Shannon–Wiener diversity index (*H*) indices, while C4S1 had the highest Pielou’s evenness index (*E*) indices [29]. It can be seen that different row ratios of corn and soybean intercropping have various impacts on insect pest control.

With the increasing demand for corn and soybeans, many countries have strongly promoted corn and soybean intercropping to prevent a decline in corn production and to achieve an additional soybean harvest [23,30]. Azizi et al. (2021) suggested that the corn yield under CSSI system was greater than that of monocropped [31]. Weeding and physical insect control under CSSI systems could prevent possible yield loss [31]. It is clear from the study that intercropping planting led to a decrease in the number of aphids on soybean plants and could prevent possible yield loss [32]. Analysis revealed that corn and soybean yield were significantly affected by planting density, strip width, and the number of rows [30,33]. Particularly, soybean yield was affected by the strip distance and the row number; when the planting density was higher, shading occured which caused lower yield than that of monoculture crops in the CSSI system [33]. Certain corn and soybean intercropping patterns have been found to negatively impact the yield and quality of soybean crops [18,23]. And the 2:2 row ratio of corn and soybean intercropping was identified as the most effective for maximizing system yield [23]. Li et al. (2022) proved that the 2:2 row ratio of corn and soybean intercropping effectively reduced the abundance of insect pests and maintained soybean yield in the CSSI system [18]. Deng et al. (2024) showed that expanding the bandwidth could improve the light environment for corn, boost light transmission, and maximize soybean leaf photosynthetic rate [30]. The land use efficiency (*LER*) of all eight corn and soybean intercropping patterns was more than 1, and the C3S3 pattern had the highest *LER* [30]. Compared with the C2S4 and C4S4 patterns, corn plants under the C3S4 pattern exhibited poorer ventilation and light penetration, and smaller agronomic indices [34]. Among the 2:3, 3:3, and 4:3 patterns, the 4:3 pattern exhibited the lowest soybean yield, and the 3:3 pattern exhibited an intermediate soybean yield and the lowest system yield [35]. Zhan et al. (2019) conducted a global meta-analysis of 47 studies reported in England and 43 studies reported in China to assess land use efficiency (i.e., *LER*) in corn and soybean intercropping as compared to monoculture crop, it was concluded that the worldwide average land equivalent ratio (*LER*) of the CSSI system was 1.32 ± 0.02 [33].

The CSSI system can enhance ecological control services of insect pests and system production, which is a sustainable cropping system worldwide [23]. But meta-analyses predominantly focus on singular aspects, such as pest control [3], or water and nitrogen fertilization [33], neglecting a holistic evaluation about pest control and system yield. Moreover, few existing analyses have systematically explored the effects of the C3S3 and C3S4 patterns on pest control and yield. To address these gaps, we conducted a two-year field experiment to evaluate the impact of the CSSI system on ecological insect pest control and yield of the 3:3 and 3:4. Specifically, the following questions were posed: (1) Does the CSSI system drive insect community diversity? (2) Does the CSSI system reduce the abundance of total insect pests or key insect pests? (3) Does the CSSI system enhance the land use and system crop yield?

## 2. Results

### 2.1. Impacts of the Corn–Soybean Strip Intercropping (CSSI) on Community Components of Insect Pests

Based on the number of insect pests found, the key insect pests on corn and soybean plants in the CSSI systems and monoculture system (including C, S, C3S3-C, C3S3-S, C3S4-C, and C3S4-S subplots) were found that *Trialeurodes vaporariorum*, *Cicadella viridis*, *Halyomorpha halys*, *Riptortus pedestris*, *Locusta migratoria manilensis*, *Spodoptera litura*, *Pyrausta nubilalis*, *Adelphocoris fasciaticollis*, Gryllulus, *Acrida cinerea*, and *Oedaleus infernalis Sauss* (Table 1). The relative abundance of *T. vaporariorum* was the highest among all the insect pests (2022: 50.2–73.4%; 2023: 59.4–68.9%), and it was the key insect pest of corn and soybean plants (Table 1).

### 2.2. Impacts of the Corn–Soybean Strip Intercropping (CSSI) on Community Diversity Indexes of Insect Pests

CSSI and sampling year significantly affected the *H* value of insect pests on corn plants (*p* < 0.01; Table 2), and CSSI also significantly influenced the value of *E* and *C* of insect pests on corn and soybean plants (*p* < 0.05, 0.01 or 0.001; Table 2), and there was significant interaction between planting pattern and sampling year on the value of *D* index of insect pests on soybean plants (*p* < 0.05; Table 2).

**Figure 1:**
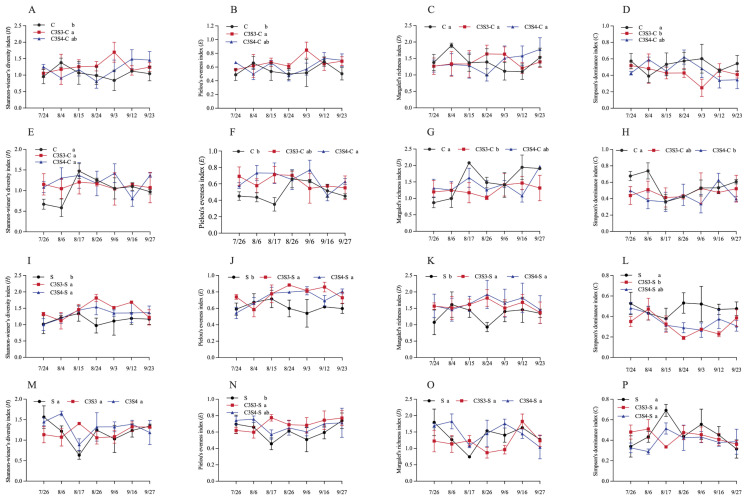
Effects of the corn–soybean strip intercropping (CSSI) system on the dynamic values of community diversity indexes of insect pests on corn (**A**–**H**) and soybean (**I**–**P**) plants over all sampling dates in 2022 (**A**–**D**,**I**–**L**) and 2023 (**E**–**H**,**M**–**P**) (Note: *H*: Shannon–Wiener’s diversity index (**A**,**E**,**I**,**M**); *E*: Pielou’s evenness index (**B**,**F**,**J**,**N**); *D*: Margalef’s richness index (**C**,**G**,**K**,**O**); *C*: Simpson’s dominance index (**D**,**H**,**L**,**P**). Different lowercase letters indicated significant differences among C, C3S3-C and C3S4-C, and among S, C3S3-S and C3S4-S by LSD test at *p* < 0.05).

For corn crops, compared with C monoculture, C3S3 significantly enhanced the values of *H* and *E* indexes of insect pests in the C3S3-C subplots in 2022 (*p* < 0.05; Figure 1A,B), and C3S4 significantly enhanced the *E* value of insect pests in the C3S4-C subplots in 2023 (*p* < 0.05; Figure 1F). Additionally, C3S3 significantly decreased the *C* value of insect pests on corn plants in C3S3-C subplots in 2022 (*p* < 0.05; Figure 1D), and C3S4 significantly decreased the values of *D* and *C* indexes of insect pests in C3S3-C and C3S4-C subplots in 2023, respectively (*p* < 0.05; Figure 1G,H).

For soybean crop, the values of *H*, *E*, and *D* index of insect pests in the C3S3-S and C3S4-S subplots were significantly higher than that in the S subplots in 2022 (*p < 0.05*; Figure 1I,J), and the *E* value of insect pests in the C3S3-S subplots were significantly higher than that in the S subplots in 2023 (*p* < 0.05; Figure 1I,J). Additionally, the *C* values of insect pests in the C3S3-S subplots were significantly lower than that in the S subplots in 2022 (*p* < 0.05; Figure 1L).

### 2.3. Effects of the Corn–Soybean Strip Intercropping (CSSI) on Population Dynamics of Total Insect Pests

The CSSI system significantly affected the abundance dynamics of total insect pests on corn plants (*p* < 0.001), and there was significant interaction between planting pattern and sampling year on the abundance dynamics of total insect pests on corn plants (*p* < 0.005; Table 2).

**Figure 2:**
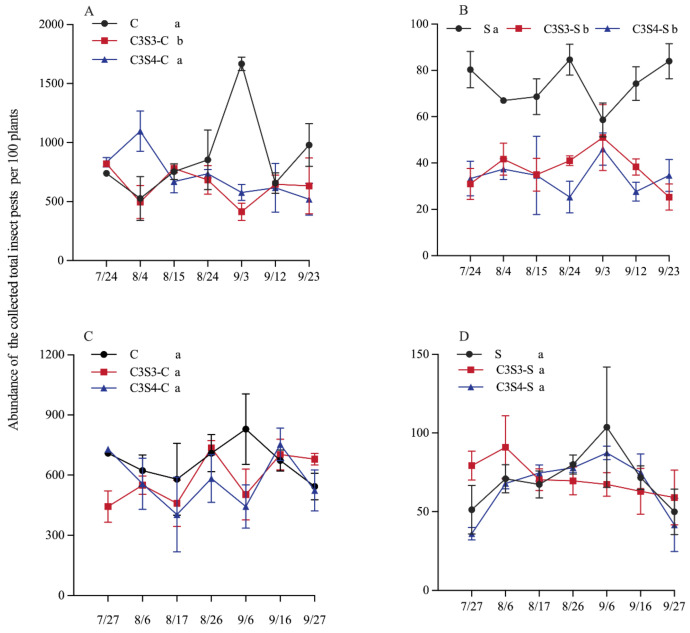
Effects of the corn–soybean strip intercropping (CSSI) system on the population dynamics of total insect pests on corn (**A**,**C**) and soybean (**B**,**D**) plants over all sampling dates in 2022 (**A**,**B**) and 2023 (**C**,**D**) (Note: Different lowercase letters indicated significant differences among C, C3S3-C and C3S4-C, and among S, C3S3-S and C3S4-S by LSD test at *p* < 0.05).

In 2022, C3S3 significantly decreased the abundance dynamics of total insect pests on corn plants in the C3S3-C subplots compared with the C monoculture (*p* < 0.05; Figure 2A), and C3S3 and C3S4 both significantly decreased the abundance dynamics of total insect pests in the subplots of C3S3-S and C3S4-S compared with the S monoculture (*p* < 0.05; Figure 2B). In 2023, there were no significant differences on the abundance dynamics of total insect pests among C, C3S3-C and C3S4-C (*p* > 0.05; Figure 2C), and among S, C3S3-S and C3S4-S (*p* > 0.05; Figure 2D).

### 2.4. Influence of the Corn–Soybean Strip Intercropping (CSSI) on Population Dynamics of T. vaporariorum

CSSI and sampling year significantly affected the population dynamics of *T. vaporariorum* on corn and soybean plants (*p* < 0.05, 0.01 or 0.001; Table 2), and there was significant interaction between planting pattern and sampling year on the population dynamics of *T. vaporariorum* on soybean plants (*p* < 0.01; Table 2).

**Figure 3:**
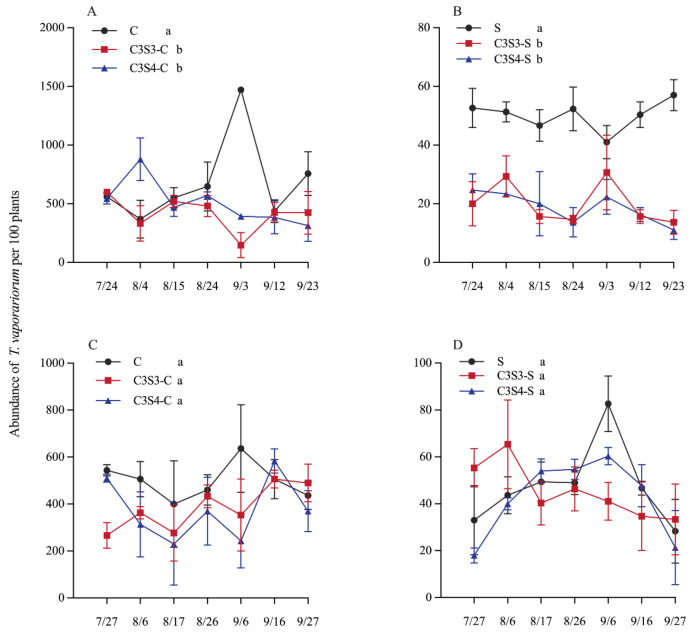
Influences of the corn–soybean strip intercropping (CSSI) system on the population dynamics of *T. vaporariorum* on corn (**A**,**C**) and soybean (**B**,**D**) plants throughout all sampling dates in 2022 (**A**,**B**) and 2023 (**C**,**D**) (Note: Different lowercase letters indicated significant differences among C, C3S3-C and C3S4-C, and among S, C3S3-S and C3S4-S by LSD test at *p* < 0.05).

In 2022, C3S3 and C3S4 significantly decreased the population dynamics of *T. vaporariorum* in the subplots of C3S3-C and C3S4-C compared with the C monoculture (*p* < 0.05; Figure 3A), and also significantly reduced the population dynamics of *T. vaporariorum* in the subplots of C3S3-S and C3S4-S compared with the S monoculture (*p* < 0.05; Figure 3B). In 2023, there were no significant differences on the population dynamics of *T. vaporariorum* among C, C3S3-C and C3S4-C (*p* > 0.05; Figure 3C), and among S, C3S3-S and C3S4-S (*p* > 0.05; Figure 3D).

### 2.5. Impacts of the Corn–Soybean Strip Intercropping (CSSI) on the Biomass and Yield Indexes of Corn and Soybean Crops

The CSSI system significantly affected the biomass, 1000-grain weight, and yield per ha of the corn and soybean crops (*p* < 0.001), and sampling year significantly influenced the values of yield indexes except of the biomass of the corn crop (*p* < 0.05, 0.01 or 0.001; Table 2). Moreover, there were significant interactions between planting pattern and sampling year on the biomass (*p* < 0.001), 1000-grain weight (*p* < 0.001), and yield per ha (*p* < 0.05) of corn crop (Table 2).

**Figure 4:**
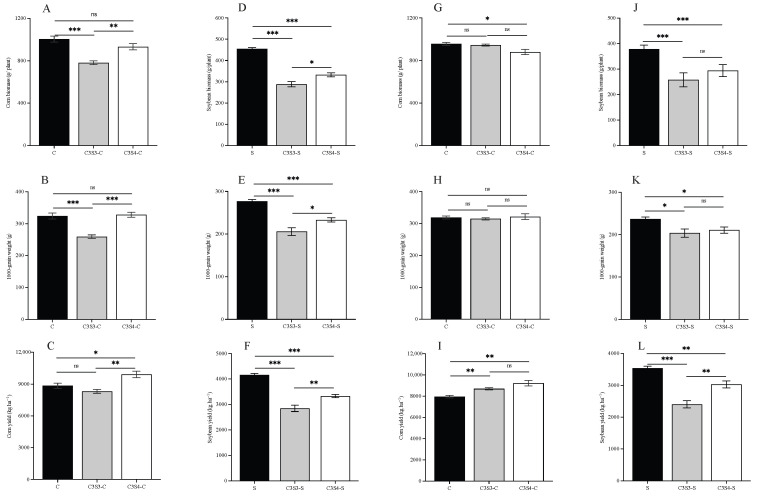
Effects of the corn–soybean strip intercropping (CSSI) system on biomass per plant (**A**,**D**,**G**,**J**), 1000-grain weight (**B**,**E**,**H**,**K**) and yield per ha (**C**,**F**,**I**,**L**) for corn (**A**–**C**,**G**–**I**) and soybean (**D**–**F**,**J**–**L**) crops in 2022 (**A**–**F**) and 2023 (**G**–**L**) (Note: *t* test was used to analyzed the significant differences among C, C3S3-C and C3S4-C, and among S, C3S3-S and C3S4-S by *t* tests; * *p* < 0.05; ** *p* < 0.01; *** *p* < 0.001; ns *p* > 0.05).

For corn crops, the biomass per plant and the 1000-grain weight in the C3S3-C subplots were significantly lower than that in the C and C3S4-C subplots in 2022 (*p* < 0.01 or 0.001; Figure 4A,B). The yield per ha in the C3S4-C subplots was significantly higher than that in the C and C3S3-C subplots in 2022 (*p* < 0.05 or 0.001; Figure 4C). And the biomass per plant in the C3S4-C subplots was significantly lower than that in the C subplots in 2023 (*p* < 0.05; Figure 4G). Moreover, the yield per ha in the C3S3-C and C3S4-C subplots were significantly higher than that in the C subplots in 2023 (*p* < 0.01; Figure 4I).

For soybean crops, the biomass per plant, the 1000-grain weight and the yield per ha in the C3S3-S and C3S4-S subplots were significantly lower than that in the S subplots in 2023 (*p* < 0.05, 0.01 or 0.001; Figure 4D–F,J–L), and these indexes in the C3S3-S subplots were also significantly lower than that in the C3S4-S subplots in 2022 (*p* < 0.05 or 0.01; Figure 4D–F). Moreover, the yield per ha in the C3S3-S subplots was significantly lower than that in the C3S4-S subplots (*p* < 0.01; Figure 4L).

### 2.6. Impacts of the Corn–Soybean Strip Intercropping (CSSI) on the LER and CR of Corn and Soybean Crops

The corn and soybean yield in each subplot were calculated based on the yield per unit and the actual proportion of arable land allocated in the CSSI system. We further examined the effects of CSSI on the land equivalent ratio (*LER*) and the competitive ratio (*CR*) of corn and soybean crops. In 2022–2023, the *LER* value was 0.852–0.886 in the C3S3 plots, and it was 0.963–0.987 in the C3S4 plots (Table 3). Simultaneously, the *CR* value of corn crops in the C3S3-C and C3S4-Csubplots was more than 1, and that of soybean crops in the C3S3-S and C3S4-S subplots was less than 1 (Table 3), demonstrating the dominance of corn crops in the CSSI system.

### 2.7. Habitat Selection Index of Total Insect Pests and T. vaporariorum Between Corn and Soybean Plants in the Corn–Soybean Strip Intercropping (CSSI)

**Figure 5:**
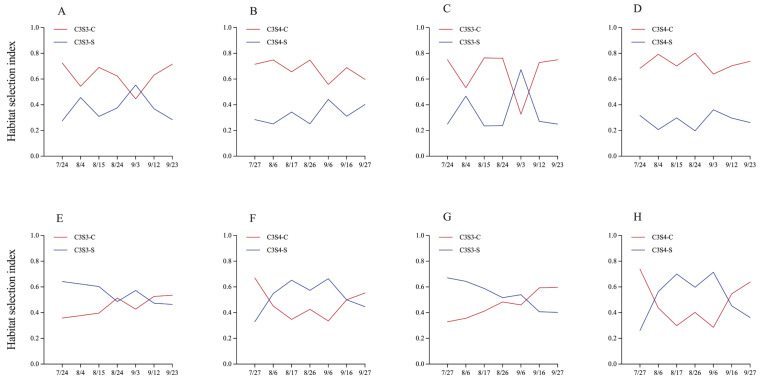
The habitat selection index of total insect pests (**A**,**B**,**E**,**F**) and *T. vaporariorum* (**C**,**D**,**G**,**H**) between corn and soybean plants in the corn–soybean strip intercropping (CSSI) system in 2022 (**A**–**D**) and 2023 (**E**–**H**) (Note: C3S3: **A**,**C**,**E**,**G**; C3S4: **B**,**D**,**F**,**H**).

In 2022, the habitat selection index of total insect pests between corn and soybean plants was more than 0.5 in the plots of C3S3 (0.625) and C3S4 (0.673), respectively (Figure 5A,B). In 2023, the habitat selection index of total insect pests between corn and soybean plants was less than 0.5 in the plots of C3S3 (0.448) and C3S4 (0.47), throughout all sampling dates (Figure 5E,F). For the key insect pest *T. vaporariorum*, the habitat selection index between corn and soybean plants was more than 0.5 in the plots of C3S3 (0.659) and C3S4 (0.723) in 2022 respectively (Figure 5C,D). And the habitat selection index of *T. vaporariorum* between corn and soybean plants both were equal to 0.5 in the plots of C3S3 (0.538) and C3S4 (0.522) throughout all sampling dates in 2023 (Figure 5G,H).

### 2.8. Relationship Between the Crop Yield and the Average Abundances of Total Insect Pests and T. vaporariorum

**Figure 6:**
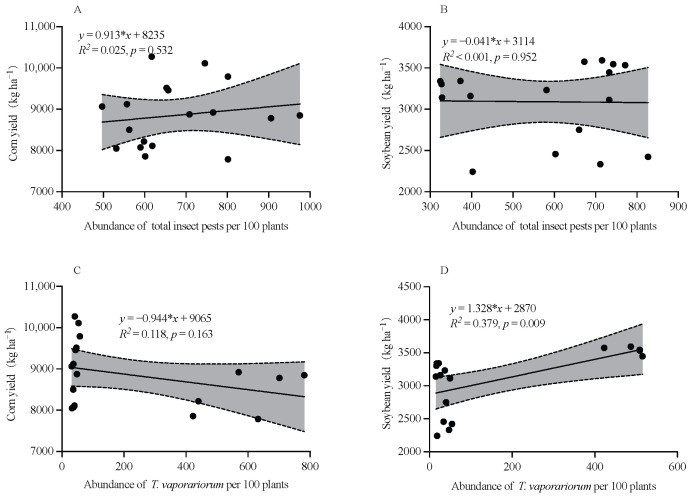
Pearson correlation test on the relationship between the yield of corn (**A**,**C**) and soybean (**B**,**D**) crops, and average abundances of total insect pests (**A**,**B**) and the key insect pest *T. vaporariorum* (**C**,**D**) in the corn–soybean strip intercropping (CSSI) system during two successive years of 2022 and 2023. (Note: The dots represented the sample number, and the lines represented the linear fit; the dotted lines and shadows indicated the 95% confidence band).

During the two successive years of 2022 and 2023, the yield of corn and soybean crops were not significantly correlated with the average abundance of total insect pests (Corn: *R*^2^ = 0.025, *p* = 0.532; Soybean: *R*^2^ < 0.013, *p* = 0.952; Figure 6A,B), and the corn yield was not significantly correlated with the average abundance of *T. vaporariorum* (*R*^2^ = 0.118, *p* = 0.163; Figure 6C), while the soybean yield was significantly positively correlated with the average abundance of *T. vaporariorum* (*R*^2^ = 0.379, *p* = 0.009; Figure 6D).

## 3. Discussion

### 3.1. Impacts of Corn–Soybean Strip Intercropping (CSSI) on Dynamic Value of the Insect Community Diversity Indexes of Insect Pests

Increasing crop biodiversity can promote natural pest control, and strip intercropping is one of the most common practices to increase crop diversity in agricultural production. Intercropping systems reduced the abundance of insect pests, while it did not affect their species richness [29]. Our findings were consistent with this conclusion. A total of 11 insect pest species were found in corn and soybean monoculture systems (i.e., C and S), also in the CSSI system (i.e., C3S3 and C3S4), and there was no significant difference in Margalef’s richness index (*D*) between strip intercropping and monoculture systems. Li et al. (2022) indicated that the impact of the CSSI system significantly reduced Simpson’s dominance index (*C*) of insect pests on soybean plants [17]. Similarly, compared with C subplots, it significantly reduced in C3S3-C subplots on corn plants in 2022; compared with S subplots, it significantly reduced both in C3S3-S and C3S4-S subplots on soybean plants in 2022. Li et al. (2022) indicated that the value of the Shannon–Wiener diversity index (*H*), and the Pielou’s evenness index (*E*) were significantly higher in the CSSI system than that in corn monoculture on corn plants [18]. We also observed the phenomenon. Compared with the monoculture systems (i.e., C and S), the values of the Shannon–Wiener diversity index (*H*), Pielou’s evenness index (*E*), and Margalef’s richness index (*D*) indexes were relatively higher, while the Simpson’s dominance index (*C*) value was lower in the CSSI system (i.e., C3S3 and C3S4). Li et al. (2019) indicated that the values of the Shannon–Wiener diversity index (*H*), Pielou’s evenness index (*E*), and Margalef’s richness index (*D*) indexes in the CSSI system were all higher than that in monoculture systems, while the Simpson’s dominance index (*C*) value in monoculture systems was higher than that in the CSSI system [25]. Our results indicate that the CSSI system is conducive to the community stability of the insect community and is not favorable for the outbreak of dominant insect pests [25].

### 3.2. Impacts of Corn–Soybean Strip Intercropping (CSSI) on Population Dynamics of Total Insect Pests

In agroecosystems, natural pest control from “top-down” and “bottom-up” processes of crop plants limits pest impacts on crops [2,36]. Many studies indicated that the CSSI system can hold promise for natural control services [3,18,19]. Our results showed that the C3S3 and C3S4 patterns suppressed the population dynamics of total insect pests on corn plants. In the C3S3-C and C3S4-C subplots, the population dynamics of total insect pests decreased by 12.71–27.58% and 14.49–18.24% compared with that in the C subplots in 2022–2023, respectively; and that in the C3S3-S and C3S4-S subplots decreased by 5.26–49.13% and 5.86–53.83% compared with those in the C subplots in 2022–2023, respectively. Our results showed that the CSSI system had a significant effect on the control of insect pests on both soybean and corn crops. The meta-analysis indicated that the intercropping pattern cut arthropod pest abundance by 38% and density by 41% [3]. Ju et al. (2019) investigated the impact of corn and peanut (*Arachis hypogaea*) strip intercropping on natural enemy populations, noting a substantial increase in the abundance of ladybird beetle and a reduction in abundance of *Aphis craccivora* on peanut plants [2]. Moreover, they found that ladybird beetles preferred to inhabit corn plants. It indicated that in the corn–peanut strip intercropping system, corn plants provided higher-quality habitats for these natural enemies and influenced the pests control on the peanut crops [2]. On the other hand, richer crop mixes in intercropping boost volatile diversity, raising insect diversity and suppressing insect pests, and the volatile compounds released by plants have repellent effects on insect pests [37]. For instance, the tetradecanoic acid and hexadecanoic acid released from the leaves of corn had a significant repellent effect on the orientation of *Ostrinia furnacalis* [9,10]. In strip intercropping systems, push-pull systems employ chemically repellent intercrops to drive pest insects away from the main cash crop [11]. Meanwhile, a more chemically attractive host species is planted nearby to attract insect pests in crop fields. Fyie et al. (2025) indicated that the silflower–sweetclover and silflower–wheat strip intercropping modified plant growth and VOC production of these crop plants, potentially influencing their pest defense when they were grown in more biodiverse assemblages [38]. In our work, further work will need to continuously track the occurrence of natural enemies and insect pests in the C3S3 and C3S4 patterns. Moreover, future research needs to identify the specific chemical substances released by one crop when it is attacked by pests in the CSSI system, which can be perceived by the other crop in the intercropping arrangement to enhance its defense capability against pests.

### 3.3. Impacts of Corn–Soybean Strip Intercropping (CSSI) on Population Dynamics of T. vaporariorum

Intercropping systems are chiefly implemented to exert targeted suppression on the key pest species in agroecosystems [18,36,39]. The population of *Nezara viridula* pests was lower in the CSSI system (C3S3 and C3S4) compared to soybean monocultures [28]. Saied et al. (2024) indicated that intercropping patterns led to a decrease in aphid (the key insect pest) populations on soybean plants compared to soybean monoculture [32].We evaluated the influence of intercropping patterns on the population dynamics of *T. vaporariorum* to further evaluate the ecological control of the CSSI system. Similarly, there was significant difference in the average abundance of *T. vaporariorum* on corn or soybean plants among different subplots (C, C3S3-C, and C3S4-C subplots; S, C3S3-S, and C3S4-S subplots) in 2022. Compared with the corn (C) and soybean (S) monoculture, the CSSI system (i.e., C3S3 and C3S4) reduced the population dynamics of *T. vaporariorum* on corn plants by 22.923–38.831% and 24.928–25.818%, and that on soybean plants decreased by 4.910–60.247% and 11.022–62.619% from 2022 to 2023. Li et al. (2022) found that the strip intercropping decreased the abundance of the key pest species *O. furnacalis* on corn plants [18]. The intercropping corn with cowpea reduced the larval population abundance of *S. frugiperda* by 24.54–30% on corn plants [40]. And the population of *T. vaporariorum* in the C2S3 pattern was the highest [19]. Fattah et al. (2023) showed that the average population of *Aphis glycines* had the maximum population in soybean monoculture and the lowest in the C2S4 and C2S6 patterns [22]. Therefore, the CSSI system significantly enhanced ecological control of insect pests. Intercropping can reduce pest pressure versus monocultures by blocking, hiding, and confusing host-seeking insects [2,8,36]. In the intercropping systems of *T. aestivum* L. and *Trifolium repens* L., aphids took more time to locate their host plants and then spent less time on wheat when it was intercropped with *T. repens* [8]. In the corn–peanut strip intercropping, more than 90% of ladybird beetles preferred to inhabit corn plants, and less than 10% of ladybird beetles inhabited peanut plants [2]. In intercropping fields, crop diversity disrupts the uniform environment on which insect pests rely for feeding and reproduction, reducing target pest incidence by 20–40% [36]. In the CSSI systems, the manipulation of corn and soybean row ratios reconfigures the spatial heterogeneity of host-plant availability, thereby disrupting the aggregation behavior of insect pests and diluting their feeding pressure below the economic injury level. In this study, the result of habitat selection index of *T. vaporariorum* showed that it preferred to inhabit corn plants both in the C3S3 (0.538–0.659) and C3S4 (0.522–0.723) patterns throughout all sampling dates in 2022–2023. As the abundance of insect pests increases, crop damage intensifies and yield losses become more severe [2]. In this study, the linear regression between population abundance of *T. vaporariorum* and crop yield did not reach statistical significance.

### 3.4. Impacts of Corn–Soybean Strip Intercropping (CSSI) on the Yield of Corn and Soybean Crops

In terms of CSSI system ecological control of insect pests, yield losses remain a key concern in corn and soybean intercropping. The CSSI system holds promise for insect pest control and corn production, but it often reduces soybean yield, these align with findings from many previous studies [19,23,41]. Farm microclimate affects crop growth and yield. In the CSSI system, as crop rows increased, crop temperature, humidity, photosynthetic performance, and yield changed. Resource competition in the CSSI system can lead to the risk of reducing overall system yield. Optimizing row ratios is essential to minimize crop competition in the CSSI systems.

This study has indicated that soybeans have a weaker competitive ability, which led to a decline in the quality of photosynthesis in soybeans and consequently had a negative impact on its biomass and yield in the CSSI system [18,35]. And some studies have suggested that the 2:4 row ratio was optimal for soybean yield in intercropping with corn [19,34]. The 3:4 and 3:6 patterns gave the maximum grain yield of corn and soybean, respectively. In this study, the C3S4 pattern significantly increased the corn yield per hectare in 2022–2023, the CSSI system resulted in a significant reduction in soybean yield per hectare in 2022–2023, and the soybean yield in C3S4-S subplots was higher than that in the C3S3-S subplots in 2023. Wang (2022) showed that both C4S6 and C4S3 pattern resulted in a significant reduction in soybean yield [35]. The CSSI system (the C3S3, C3S4, and C3S6 patterns) resulted in a 56.70% to 77.39% reduction in soybean yield per unit area compared to the soybean monoculture pattern [28]. Similarly, the 3:3 and 3:4 patterns both decreased the soybean yield per hectare in our study. And compared to the S pattern, the C3S3 pattern significantly reduced soybean yield in 2023. Moreover, farmers usually pay more attention to the total amount of harvested yield in farmland rather than just assessing the yield per unit [36]. Our results indicated that regardless of corn or soybean crop, the actual total yield of both crops under strip intercropping fell by nearly half compared to monoculture in 2022–2023, due to their reduced planting area. A meta-analysis has showed that the total yield of intercropping corn and soybean in China increased by approximately 12%, while the equivalent yield decreased by 8% and 40% compared to monoculture, respectively [23]. Additionally, *LER* reflects the yield of two species in strip intercropping compared with monoculture. The worldwide average *LER* of the CSSI system is 1.32 ± 0.02, and that is 1.35 ± 0.03 in China, indicating a substantial land sparing potential of intercropping over monoculture crops [33]. While the CSSI systems generally enhance land use efficiency across different regions of the world, not all CSSI systems necessarily lead to an increase in *LER* [41,42]. In the same area, the *LER* of the C4S6 pattern was greater than 1, while that of C4S3 was less than 1 [35]. In C2S3 and C2S4 patterns, the *LER* decreased by 3.31 and 0.86%, the *CR* value of corn decreased by 18.04 and 24.84%, and the *CR* value of soybeans increased by 17.32 and 22.77%, respectively [30]. In this study, the partial *LER* of corn and soybean under the CSSI system were less than 1, and the total *LER* under the CSSI system were also less than 1. The *LER* of the C3S4 pattern was greater than that of the C3S3 pattern. The C3S4 pattern had a greater substantial land sparing potential than C3S3. But Hafid et al. (2021) showed that the *LER* of C3S3 and C3S4 patterns both >1 [28].

Environmental temperature is one crucial variable influencing the yield of corn and soybean crops in the CSSI system in China. The annual mean temperature contributed 5% to the *LER*, while the annual mean accumulated temperature (>10 °C) accounted for less than 4% of the *LER* [5]. The varying climates in different regions and the diverse strip ratios of corn to soybean in CSSI system can both influence *LER*. Moreover, the *CR* value of corn in the CSSI system was more than 1, and that of soybean was less than 1. So, soybeans are less competitive than corn in the CSSI system, resulting in reduced soybean yield. Our results demonstrated that the land use efficiency of the C3S3 and C3S4 is lower than that of monoculture, indicating that the CSSI system had a risk of system total crop yield loss, despite the increased in yield per hectare.

## 4. Materials and Methods

### 4.1. Experimental Site Description

The experiment was carried out in a 0.4725 ha (175 × 27 m/10,000) experimental field from 2022 to 2023 at District, Jinan City, Shandong Province of China (36°58′ N, 117°13′ E). The experimental site is located where the major farming pattern is wheat–corn rotation. In 2022–2023, the average daily temperature was 22.9 °C and 24.4 °C, and the precipitation was 81.64 and 63.32 during the reproductive period from June to October, respectively (seen in Figure S1). The experiment field located in a wheat–corn rotation area with consistent soil characteristics. From 2022 to 2023, after the winter wheat was harvested, we conducted the field trial. The basic fertility parameters of the experiment field were in Table S1. The fertilizer application in the experiment was the same as that used by local farmers for corn and soybean (N: P_2_O_5_: K_2_O = 15:15:15, 600 kg ha^−1^). Before corn and soybeans sowing, a compound fertilizer was applied to the field while plowing the soil. We bought corn (cv. NongDa 372) and soybean (cv. JiHuang 34) seeds from a local seed supplier and planted them on 10 June 2022 and 2023. The experiment field was managed like local farms, but no pesticides were used during whole growing season.

### 4.2. Experimental Treatment Setup

**Figure 7:**
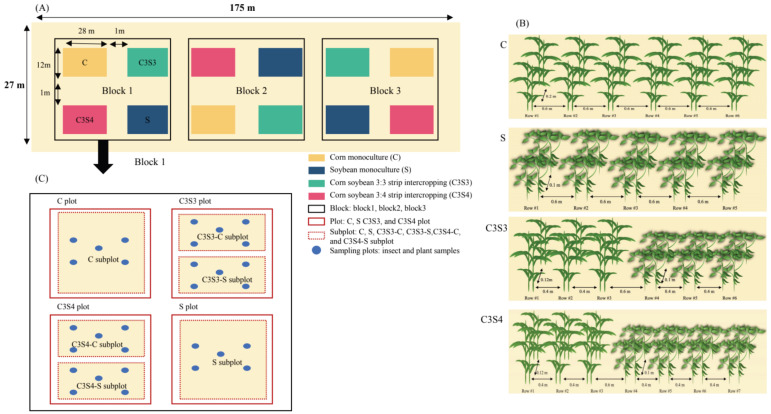
Field spatial layout of the corn–soybean strip intercropping (CSSI) system in 2022–2023. (**A**) Three blocks; (**B**) Four treatments of the monoculture of corn (C) and soybean (S), and two CSSI patterns (i.e., C3S3 and C3S4, indicating three rows of corn strip intercropped with three and four rows of soybean, respectively); (**C**) Four treatments in one block (Note: In C and S plots, each plot was manually divided into one C and one S subplot, respectively. Each C3S3 plot was manually divided into one C3S3-C and one C3S3-S subplot, and each C3S4 plot was manually divided into one C3S4-C and one C3S4-S subplot. The same in the following figures and tables).

The experiment employed a completely randomized block design with four treatments of corn monoculture (C), soybean monoculture (S), and two patterns of corn–soybean strip intercropping (i.e., C3S3 and C3S4, indicating three rows of corn strip intercropped with three and four rows of soybeans respectively), and each treatment had three replications in the experimental field which was divided into three field blocks and each block was divided into four plots (i.e., 3 plots per treatment and total 12 plots for total 4 treatments). Each block and each plot were separated by a one-meter-wide isolation belt, and each plot was 28 m length with 12 m width (i.e., 336 m^2^). For the plots of C and S treatments, the row space for both corn and soybean plants was 0.6 m, and the plant distance was 0.2 m for corn and 0.1 m for soybean, respectively. For the plots of C3S3 and C3S4, the row space for both corn and soybean plants was 0.4 m, while the row space between corn and soybean plants was 0.6 m, and the plant distance was 0.2 m for corn and 0.1 m for soybeans, respectively (Figure 7). In each C and S subplot, there were 21 rows of corn (5922 plants) and 21 rows of soybeans (11,802 plants); in each C3S3 plot, there were 15 rows of corn (4230 plants) and 15 rows of soybeans (8430 plants); in each C3S4 plot, there were 12 rows of corn (a total of 3384 plants) and 16 rows of soybeans (8992 plants).

### 4.3. Investigation and Sampling of Insect Pests

In 2022–2023, the abundance of insect pests was counted via visual observation at 10 d intervals, starting in early July and continuing until late September during the growing season of corn and soybean crops. The field investigation of insect pests was conducted by the same group of people, with no more than three individuals involved. Each plant was observed for about 4 min, and the surveys were conducted during the growing season from 7:30 a.m. to 11:30 a.m. and from 3:00 p.m. to 5:30 p.m. The investigation of insect pests was conducted seven times each year. The five-point sampling method was used to count the abundance of insect pests on the corn and soybean plants of the four treatments, i.e., C, S, C3S3 (including two subplots of C3S3-C and C3S3-S) and C3S4 (including two subplots of C3S4-C and C3S4-S) (Figure 7C). Five points were randomly selected in each subplot, and each point (0.06 × 0 2 m) included two adjacent corn or soybean plants. In each C, C3S3-C, and C3S4-C subplot, 10 corn plants were fully surveyed; in each S, C3S3-S, and C3S4-S subplot, 10 soybean plants were fully surveyed. (Figure 7C). A total of 90 corn plants and 90 soybean plants were surveyed in this study. Because *Trialeurodes vaporariorum* was the key insect pest on corn and soybean plants, it was selected to assess the ecological control of CSSI on the occurrence of key insect pests.

### 4.4. Biomass and Yield of Corn and Soybean Crops

The harvest of corn and soybean crops was carried out during the 6 and 8 October 2022 and 2023. To measure the aerial biomass (biomass) and grain yield of corn and soybean crops, 10 plant samples were randomly collected from each subplot. After cutting plants from the field, plants were separated into roots, soybean pods/corn cobs, and biomass. All samples were sun-dried to a constant weight before threshing. Later, the biomass and grain yield of each crop plant were measured by a balance with accuracy of 0.1 g and range of 0–5 kg (Shanghai Yaohua Weighing System Co., Ltd., Shanghai, China). And 1000 seeds of corn and soybean crops were randomly selected from each subplot to measure the 1000-grain weight using the same balance. The biomass and grain yield of each crop plant, and the 1000 seeds were measured with balances with accuracies of 0.1 g. Moreover, the yield per ha was obtained according to the following formula:

Yield (kg/ha) = seed weight per plant (kg) × plant number/m^2^ × 10,000 m^2^/ha (1)

### 4.5. Measured Indexes of Insect Pests on Corn and Soybean Crops

#### 4.5.1. Community Diversity Indexes of Insect Pests

The community diversity indexes of insect pests (including Shannon–Wiener index (*H*), Pielou’s evenness index (*E*), Margalef’s richness index (*D*), and Simpson’s dominance index (C)) were calculated based on the species and abundance of insect pests collected from each subplot of the four treatments, i.e., C, S, C3S3 (including two subplots of C3S3-C and C3S3-S) and C3S4 (including two subplots of C3S4-C and C3S4-S). The formulas were following as:

(2) $$\text{Shannon–Wiener}\;\mathrm{index}:\;H=\sum_{i=1}^{s}P_{i}\times \ln\;(P_{i});\;P_{i}\;=\;N_{i}/N$$

Pielou’s evenness index: *E = H/H_max_*; *H_max_ =* ln *S* (3)

Margalef’s richness index: *D* = (S − 1)/ln *N* (4)

(5) $$\text{Simpson’s dominance index}:\;C=\sum_{i=1}^{s}\;{\left(P_{i}\right)}^{2};\;P_{i}\;=\;N_{i}/N$$

Here, *N_i_* was the number of individuals for the species *i*; *N* was the total number of individuals of all species in the community of insect pests; *S* was the number of species in the community of insect pests; *H_max_* was the maximum diversity index *H*.

#### 4.5.2. Habitat Selection of Total Insect Pests and Key Insect Pest *T. vaporariorum*

The habitat selections of total insect pests and key insect pest *T. vaporariorum* between corn and soybean were evaluated in the C3S3 (C3S3-C and C3S3-S) and C3S4 (C3S4-C and C3S4-S) patterns to further clarify the ecological control of the CSSI system on insect pests of corn and soybean crops. The habitat selection index of total insect pests and *T. vaporariorum* was calculated based on their abundance in each subplot in the same plot. The investigation method for insect pest number was conducted as previously described, and the data come from the Section 4.3. investigation and sampling of insect pests. The formula is as follows:

Habitat selection index = Abundance of insect pests on corn    plants/(abundances of insect pests on corn and soybean plants)(6)

This habitat selection index revealed the distribution preference of total insect pests and *T. vaporariorum* to corn and soybean plants; an index value above 0.5 indicated a preference for corn plants, while an index value below 0.5 suggested a preference for soybean plants, and an index value of exactly 0.5 denoted no habitat selectivity.

### 4.6. LER and CR of Corn and Soybean Crops Under the Strip Intercropping

#### 4.6.1. LER Calculation

The land equivalent ratio (*LER*) reflected the yield of two types of crops (corn and soybean) in the strip intercropping relative to monoculture of corn and soybean crops. The *LER* was calculated as below [42]:

*LER* = *LER*_*c*_ + *LER*_*s*_ (7)

*LER*_*c*_ = *Y_ic_/Y_c_* (8)

*LER_s_* = *Y_is_/Y_s_* (9)

Firstly, Formula (1) was utilized to calculate the average yield per hectare of corn and soybean under different planting patterns. Subsequently, theoretical yields were ascertained by evaluating the proportion of the plot area dedicated to corn and soybean relative to the overall area. Ultimately, the pertinent performance metrics were derived through the application of Formulas (7)–(9). Here, *LER_c_* and *LER_s_* were partial *LER* of corn and soybean crops, respectively. The yield of monoculture corn and the intercropped corn were represented as *Y_c_* and *Y_ic_*, respectively. And the yield of monoculture soybean and the intercropped soybean were represented as *Y_s_* and *Y_is_*, respectively.

#### 4.6.2. CR Calculation

The competition between corn and soybean crops in the strip intercropping system was represented by the competitive ratio (*CR*), a higher *CR* value indicated a competitive advantage of the dominant crop in an intercropping system, and the *CR* was calculated as below [42]:

*CR_c_* = (*LER_c_*/*LER_s_*) × (*P_is_*/*P_ic_*) (10)

*CR_s_* = (*LER_s_*/*LER_c_*) × (*P_ic_*/*P_is_*) (11)

*LER_c_* and *LER_s_* as previously described. The proportions of land occupancy of corn and soybean crops in the strip intercropping system were represented as *P_ic_* and *P_is_*, respectively.

### 4.7. Data Analysis

SPSS software version 21.0 package (IBM, Armonk, NY, USA) and Graph Pad 9.5.0 (Graph Pad Software Inc., La Jolla, CA, USA) were used for the statistical analysis. Each subplot represented one replication level. All ecological and yield indices had three replications. Two-way repeated measures ANOVAs in SPSS software version 21 were also used to analyze the effects of sampling year (2022 vs. 2023), planting pattern (C, C3S3-C and C3S4-C; S, C3S3-S and C3S4-S) and their interaction (with sampling time as repeated measures) on abundance dynamics of total insect pests and key insect pest *T. vaporariorum*, and the value dynamics of community diversity indexes of insect pests (*H*, *E*, *D* and *C*); the *LSD* test was used to analyze the significant differences among C, C3S3-C and C3S4-C, and among S, C3S3-S and C3S4-S at *p* < 0.05, using SPSS software version 21. Moreover, two-way ANOVAs were used to analyze the effects of sampling year, planting pattern and their interaction on the biomass per plant, 1000-grain weight and the yield of corn and soybean, and *t* tests were used to analyze the significant differences among C, C3S3-C and C3S4-C, and among S, C3S3-S and C3S4-S in same sampling year, using SPSS software version 21. The habitat selection figures were created by Graph Pad version 9.5.0. The data analysis and visualization specifically involved performing “Contingency Table analyses” with the method of calculating the “Fraction of Total”. In addition, the Pearson correlation test by SPSS software version 21 was also used to detect the relationship between the crop yield and the average abundances of total insect pests and key insect pest *T. vaporariorum*. If the relationship was significant, two separate linear regression analyses were conducted to explore the relationships between the abundance of total insect pests or *T. vaporariorum* and the yields of corn or soybean crops, respectively. In two regression models, the abundance of total insect pests or *T. vaporariorum* was utilized as the predictor (*x*), and the yields of corn and soybean crops as the response (*y*), respectively. All figures were made by Graph Pad version 9.5.0.

## 5. Conclusions

This study, for the first time, explored the insect pest population dynamics, crop yields, and the *LER* under corn and soybean monoculture, and the C3S3 and C3S4 patterns. A two-year field trial demonstrated that the CSSI system enhanced the ecological control services of insect pests on corn and soybean crops. It effectively reduced the population dynamics of total insect pests, and significantly decreased the population dynamics of the key insect pest *T. vaporariorum* on corn and soybean plants, so that the CSSI system is conducive to the community stability of insect pests, and it is not favorable for the outbreak of key insect pests on corn and soybean crops. Moreover, the CSSI system increased the corn yield per hectare, but it had a negative impact on the soybean yield per hectare. However, the *LER* of C3S3 and C3S4 patterns were less than 1, indicating a potential risk of crop yield loss faced by strip intercropping. Choosing the appropriate raw ratios based on the local climate is crucial for ensuring soybean yields. The soybean yields in particular were significantly influenced by the raw ration of corn and soybeans, compared to corn yields in the CSSI system. The results of this study provide a piece of overall information that the CSSI system, especially the C3S3 and C3S4 patterns, may serve as alternatives to the corn and soybean monoculture patterns in this area. The *CSSI* system demonstrated effective pest control and increased corn yield per hectare, although it reduced soybean yield per hectare and the *LER*. The CSSI system, particularly the C3S4 pattern, may be applicable to other temperate regions with similar agroecological conditions, especially where pest pressure is a significant challenge for corn monoculture. Also, since the *CR* of corn is greater than soybean in the CSSI system, if the economic value derived from increased corn yields can compensate for and potentially surpass the economic loss from reduced soybean yields, this would indicate a significant economic advantage. Optimizing the corn–soybean balance within the CSSI system can enhance overall productivity and contribute to sustainable intensification and food security goals, despite reduced soybean yields per hectare. Additionally, these findings need verification in different geographical contexts. We recommend conducting studies on a larger scale and in various temperate regions to ensure recommendations of the CSSI system under a range of conditions.

## Figures and Tables

**Table 1:** The relative abundance of the insect pests on corn and soybean plants in different subplots under different planting patterns over all sampling dates.

Year	Insects	Subplots					
		C	C3S3-C	C3S4-C	S	C3S3-S	C3S4-S
2022	*T. vaporariorum*	73.4%	60.4%	65.5%	68.2%	50.2%	52.0%
	*C. viridis*	6.0%	8.8%	9.2%	7.2%	7.8%	9.2%
	*H. halys*	4.8%	6.0%	6.0%	4.3%	6.3%	4.1%
	*R. pedestris*	4.4%	6.3%	4.1%	7.2%	10.8%	8.7%
	*L. migratoria manilensis*	2.0%	5.5%	4.1%	4.1%	9.1%	7.5%
	*S. litura*	3.4%	4.5%	3.4%	3.3%	9.0%	7.9%
	*P. nubilalis*	2.8%	2.1%	3.3%	2.0%	4.0%	3.0%
	*A. fasciaticollis*	1.6%	2.8%	2.0%	2.0%	0.8%	1.7%
	Gryllulus	1.2%	2.8%	1.2%	1.0%	1.4%	2.5%
	*A. cinerea*	0.3%	0.1%	1.2%	0.5%	0.6%	1.8%
	*O. infernalis sauss*	0	0.6%	0.1%	0.1%	0	0.5%
2023	*T. vaporariorum*	65.7%	64.7%	68.9%	65.6%	61.8%	59.4%
	*C. viridis*	7.7%	7.2%	8.2%	6.6%	8.7%	6.6%
	*H. halys*	3.5%	7.4%	5.7%	4.9%	2.0%	6.5%
	*R. pedestris*	6.4%	6.6%	4.5%	6.4%	10.2%	7.0%
	*L. migratoria manilensis*	4.5%	5.0%	4.3%	5.3%	4.2%	5.9%
	*S. litura*	3.1%	5.0%	3.6%	4.0%	3.3%	5.0%
	*P. nubilalis*	4.7%	1.6%	2.8%	2.8%	1.5%	3.7%
	*A. fasciaticollis*	2.1%	1.1%	1.4%	2.2%	0.9%	3.3%
	Gryllulus	1.3%	1.4%	0.3%	0.6%	0.3%	0.8%
	*A. cinerea*	0.8%	0.1%	0.3%	1.1%	0	0.7%
	*O. infernalis sauss*	0.2%	0	0	0.4%	0	1.1%

**Table 2:** Two-way repeated measures ANOVAs (with sampling time as repeated measures) about the effects of sampling year (2022 vs. 2023), planting pattern (including C, C3S3-C and C3S4-C; S, C3S3-S and C3S4-S) and their interaction on abundance dynamics of total insect pests and key insect pest *Trialeurodes vaporariorum*, and value dynamics of community diversity indexes of insect pests; and two-way ANOVAs about the effects of sampling year, planting pattern and their interaction on the biomass per plant, 1000-seed weight and yield per ha of corn and soybean crops (*F*/*p* values).

Measured Indexes	Crop	Sampling Year (Y)	Planting Pattern (*p*)	Y × *p*
Abundance of total insect pests	Corn	1.63/0.15	5.05/<0.001 ***	2.83/0.003 **
	Soybean	1.58/0.17	0.61/0.83	1.56/0.12
Shannon–Wiener index (*H*)	Corn	16.60/0.002 **	7.90/0.006 **	1.66/0.23
	Soybean	1.06/0.32	3.56/0.061	1.98/0.18
Pielou’s evenness index (*E*)	Corn	0.42/0.53	8.58/0.005 **	1.15/0.35
	Soybean	4.56/0.054	12.00/<0.001 ***	0.34/0.72
Margalef’s richness index (*D*)	Corn	0.01/0.93	2.01/0.18	2.16/0.16
	Soybean	2.72/0.13	2.30/0.14	6.17/0.014 *
Simpson’s dominance index (*C*)	Corn	0.46/0.51	6.79/0.011 *	1.05/0.38
	Soybean	1.71/0.22	6.14/0.015 *	1.95/0.18
Abundance of *T. vaporariorum*	Corn	8.44/0.013 *	8.15/0.006 **	1.20/0.34
	Soybean	29.60/<0.001 ***	16.90/<0.001 ***	10.2/0.003 **
Biomass (g/plant)	Corn	0.70/0.41	12.50/<0.001 ***	14.80/<0.001 ***
	Soybean	11.40/<0.001 ***	35.80/<0.001 ***	0.96/0.39
1000-seed weight (g)	Corn	5.71/0.019 *	13.30/<0.001 ***	9.28/<0.001 ***
	Soybean	5.22/0.025 *	11.10/<0.001 ***	1.39/0.25
Yield (kg/ha)	Corn	6.68/0.011 *	18.60/<0.001 **	4.59/0.013 *
	Soybean	11.30/<0.001 ***	28.00/<0.001 ***	0.48/0.62

Note: * *p* < 0.05; ** *p* < 0.01; *** *p* < 0.001.

**Table 3:** The land equivalent ratio (*LER*) of corn (*LER_c_*) and soybean (*LER_s_*), and the competitive ratio (*CR*) of corn (*CR_c_*) and soybean (*CR_s_*) in the corn–soybean strip intercropping (CSSI) systems in 2022–2023.

Year	Planting Pattern	Corn		Soybean		Corn + Soybean
		*LER_c_*	*CR_c_*	*LER_s_*	*CR_s_*	*LER*
2022	C3S3	0.47 ± 0.03	1.33 ± 0.43	0.38 ± 0.11	0.80 ± 0.22	0.85 ± 0.13
	C3S4	0.49 ± 0.01	1.42 ± 0.38	0.48 ± 0.11	0.73 ± 0.17	0.96 ± 0.10
2023	C3S3	0.55 ± 0.05	1.61 ± 0.16	0.34 ± 0.01	0.63 ± 0.06	0.89 ± 0.50
	C3S4	0.50 ± 0.02	1.36 ± 0.11	0.49 ± 0.05	0.74 ± 0.06	0.99 ± 0.06

## Data Availability

The original contributions presented in the studies are included in the article; further inquiries can be directed to the corresponding author.

## Supplementary Materials

The following supporting information can be downloaded at: https://www.mdpi.com/article/10.3390/plants14213358/s1, Figure S1: Average temperatures and rainfall accumulations from June to September in 2022 and 2023 during the experiment; Table S1: Soil properties in the experiment fields.
