# Supplementary material for: Effects of Corn–Soybean Strip Intercropping on Control Efficiency of Insect Pests and Crop Yields
Source: Plants (Basel). 2025 Nov 2;14(21):3358. doi: 10.3390/plants14213358 (PMC12609226; doi:10.3390/plants14213358)
Supplement: Supplementary file 1 [file plants-14-03358-s001.zip › 1-Supporting information-Chen edited.pdf]

Supporting Information

Effects of Corn-Soybean Strip Intercropping on Pest Control Efficiency and Crop Yields

Xiping Wei[1]; Zhoulong Cheng[1]; Junjie Wang[2]; Chongyi Liu[1]; Shanglin Yang[1]; Fajun Chen[1,*]

1: State Key Laboratory of Agricultural and Forestry Biosecurity, Department of Entomology, College of Plant Protection, Nanjing Agricultural University, Nanjing 210095, China; 2024202066@stu.njau.edu.cn (X.W.); chengzhoulong@stu.njau.edu.cn (Z.C.); liuchongyi@stu.njau.edu.cn (C.L.); 2022802167@stu.njau.edu.cn (S.Y.)

2: College of Agriculture, Nanjing Agricultural University, Nanjing 211800, China; 11121203@stu.njau.edu.cn (J.W.)

*: Correspondence: fajunchen@njau.edu.cn (F.C.)

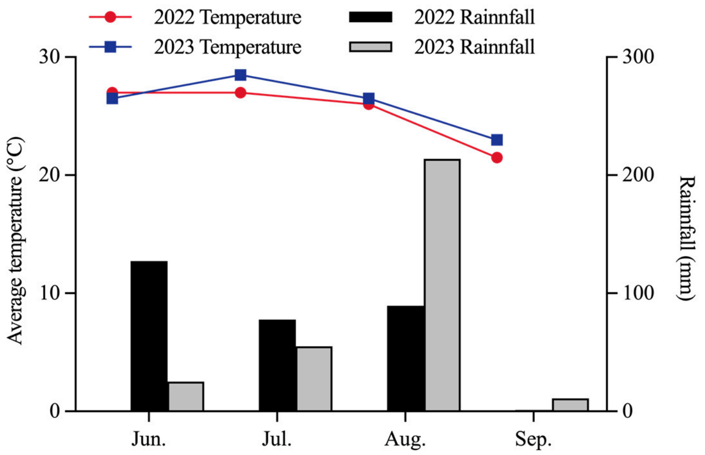

Figure S1. Average temperatures and rainfall accumulations from June to September in 2022 and 2023 during the experiment (Note: Data was from the Jiyang Statistical Yearbook of 2022 and 2023)

Table S1. Soil properties in the experiment fields

pH	Total carbon g/kg	Total nitrogen g/kg	Available phosphorus mg/kg	Available potassium mg/kg	Organic carbon g/kg
7.96	0.67	0.76	28.97	99.84	6.63
